# Treatment resistant depression in women with peripartum depression

**DOI:** 10.1186/s12884-019-2462-9

**Published:** 2019-09-02

**Authors:** M. Soledad Cepeda, David M. Kern, Susan Nicholson

**Affiliations:** 1 Janssen Research and Development, 1125 Trenton Harbourton Rd, Titusville, NJ 08560 USA; 2 Johnson & Johnson Women’s Health, New Brunswick, NJ Canada

**Keywords:** Peripartum depression, Treatment resistant depression in pregnant women, Claims database studies

## Abstract

**Background:**

Peripartum depression is a leading cause of disease burden for women and yet there is little evidence as to how often peripartum depression does not respond to treatment and becomes treatment resistant depression. We sought to determine the incidence of treatment resistant depression (TRD) in women with peripartum depression.

**Methods:**

Population based retrospective cohort study using a large US claims database. Peripartum depression was defined as having a depression diagnosis during pregnancy or up to 6 months after the end of pregnancy. We included women with prevalent or incident depression. The outcome was the development of TRD within 1 year after the diagnosis of peripartum depression. TRD was defined as having 3 distinct antidepressants or 1 antidepressant and 1 antipsychotic in 1 year.

Women with peripartum depression may not be exposed to pharmacological treatments early in pregnancy, therefore we created two groups: 1. women with peripartum depression, and 2. women with peripartum depression diagnosed 3 months before a live birth delivery or within 6 months after that delivery.

**Results:**

There were 3,207,684 pregnant women, of whom 2.5% had peripartum depression. Of these women half had incident depression during pregnancy. Five percent of women with peripartum depression developed TRD within 1 year of the depression diagnosis. The risk of developing TRD was 50% higher in women with prevalent depression than in women with incident peripartum depression (*P* < 0.0001). Results were similar in women with peripartum depression diagnosed later in their pregnancy.

Women who went on to develop TRD had more substance use disorders, anxiety, insomnia and painful conditions.

**Conclusions:**

TRD occurs in approximately 5% of women with peripartum depression. The risk of TRD is higher in pregnant women with a history of depression. Women who went on to develop TRD had more psychiatric comorbidities and painful conditions than women who did not.

**Electronic supplementary material:**

The online version of this article (10.1186/s12884-019-2462-9) contains supplementary material, which is available to authorized users.

## Introduction

Peripartum depression is not only a leading cause of disease burden for women and their families [1], but also has been associated with negative effects on the fetus, infant and young child [2]. Peripartum depression is clinically diagnosed when mood symptoms of major depression occur during pregnancy or in the 4 weeks following delivery [3, 4]. Studies have shown that a woman has a greater risk of being admitted to a psychiatric hospital within the first month postpartum than at any other time in her life [5]. Nonetheless, it has been recommended that the diagnostic criteria be expanded from 1 month to 6 months after delivery, since this entire period is a high-risk time for depression [6].

The prevalence of peripartum depression ranges from 6.0 to 20% of pregnancies depending on how the diagnosis is made, the population, country or year [6–8]. In the US, the Centers for Disease Control and Prevention estimated that in 2012, 10% of women who had a live birth had peripartum depression, which is lower than the 14% estimated in 2004 [9]. The authors theorized that better recognition of risk factors for depression and improved treatment before and during pregnancy may have contributed to the decline [10].

Contrary to the amount of evidence on the prevalence of peripartum depression, there is little evidence as to how often peripartum depression does not respond to treatment and becomes treatment resistant depression. There are many treatment resistant depression (TRD) definitions [11, 12] that range from not responding to a single treatment to not responding to sequential treatments. A recent literature search found that the most commonly used definition of TRD in subjects with major depressive disorder requires a minimum of two prior treatment failures of adequate dose and duration [13]. The treatment failure excludes adverse events. The National institute of Mental Health-funded Sequenced Treatment Alternatives to Relieve Depression (STAR*D) trial provides support for this definition, as the results showed that resistance to treatment markedly increased after the failure of 2 treatments at adequate dose and duration [14, 15]. .A population-based study using healthcare databases found that in patients with newly diagnosed depression, 10% develop TRD within one year. However, it is not known how often TRD is diagnosed in women with peripartum depression. Therefore, this study sought to determine the incidence of TRD in women with peripartum depression depending on whether they had or did not have history of depression prior to pregnancy.

## Methods

We designed a population-based retrospective cohort study. We used a large US claims database and included data from January 1, 2000 to April 30, 2018.

### Source

We used the IBM MarketScan® Commercial Database (CCAE). This is a large US claims database that includes data from individuals enrolled in employer-sponsored insurance health plans. Data include adjudicated health insurance claims (e.g., inpatient, outpatient, and outpatient pharmacy) as well as enrollment data from large employers and health plans who provide private healthcare coverage to employees, their spouses, and dependents. Data elements are outpatient pharmacy dispensing claims (coded with National Drug Codes (NDC) and inpatient and outpatient medical claims, which provide diagnosis codes (ICD-9-CM or ICD-10-CM).

### Population

We included women with peripartum depression. Although peripartum depression is clinically diagnosed when mood symptoms of major depression occur during pregnancy or in the 4 weeks following delivery [4], it has been recommended that the onset be expanded from 4 weeks to 6 months after delivery [6]. Thus, we defined peripartum depression as having depression diagnosed during pregnancy or up to 6 months after the end of pregnancy.

Depression was defined using a validated algorithm that requires the presence of two depression diagnosis codes within 1 year or a hospitalization with a depression diagnosis [16]. We included both women with a history of depression prior to pregnancy (prevalent depression) and women with newly diagnosed depression (incident depression) any time during pregnancy or up to 6 months after the end of pregnancy.

**Fig. 1:**
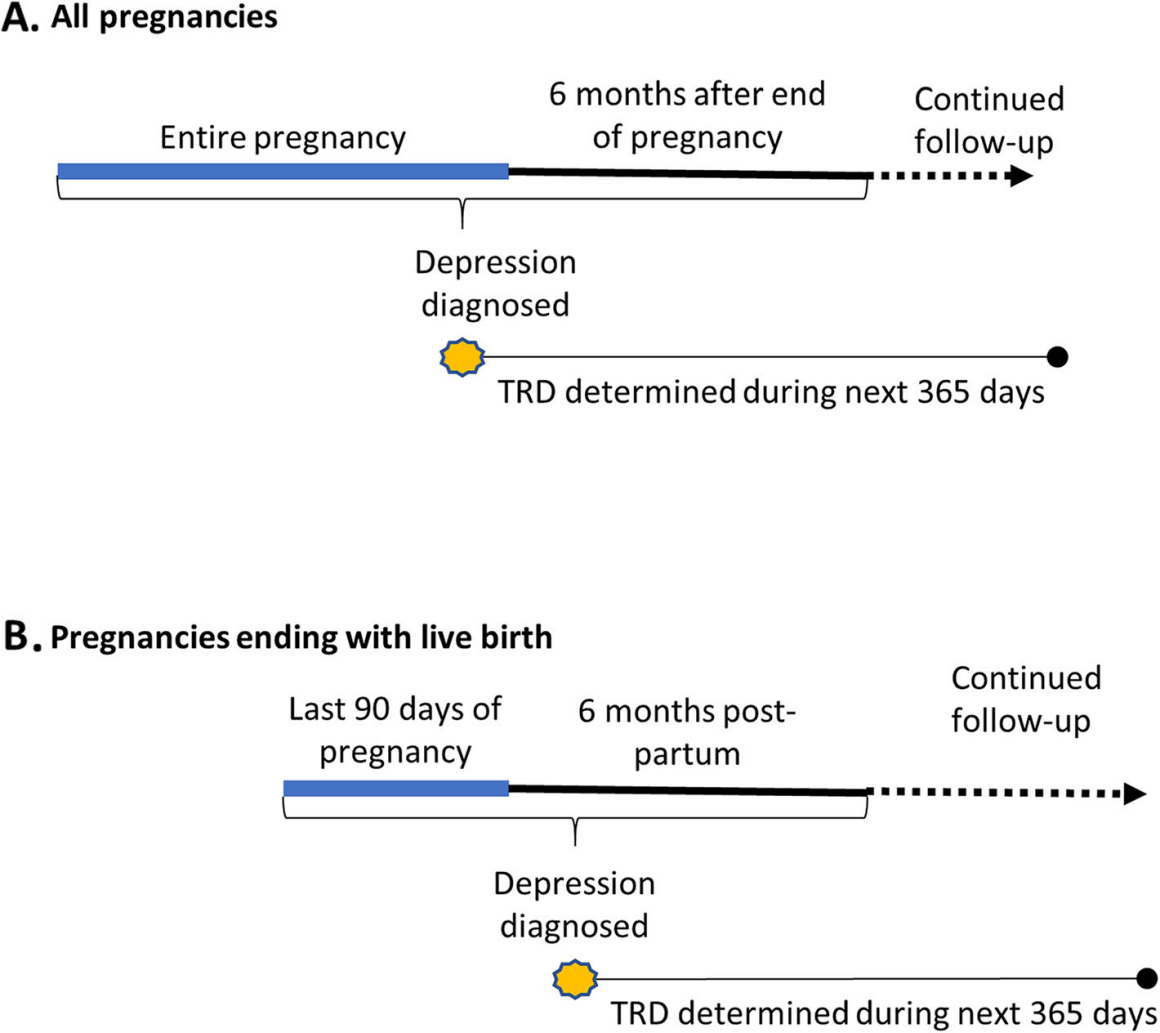
Study design illustration

Since women with peripartum depression may not be exposed to pharmacological treatments for depression early in their pregnancy because they discontinue them to conceive or after conception [17], or do not start them because of concerns of exposing the fetus to antidepressants [18], some of these women would not have the opportunity to develop treatment resistant depression, which is based on lack of response to pharmacological treatments. To address this, two groups of women were created: 1. all women diagnosed with peripartum depression; 2. only women with peripartum depression diagnosed 3 months before a live birth delivery or within 6 months after that delivery. The latter group is more likely to have the opportunity to be exposed to pharmacological treatments, even if pharmacological treatment had been postponed during early pregnancy. See Fig. 1.

Women with psychosis, bipolar disorder, or dementia before the index date (i.e., the date of the first depression diagnosis during pregnancy or 6 months after a delivery) were excluded to ensure that any antipsychotic received was for the treatment of depression and not for the treatment of psychosis. Women with bipolar disorder were excluded because when TRD is studied and defined this type of depression is often excluded [19, 20].

Pregnancy, pregnancy duration, and pregnancy outcomes were defined in the database using a validated algorithm developed for claims database studies [21]. The Systematized Nomenclature of Medicine-Clinical Terms (SNOMED) concepts used to identity depression were depression and major depression, excluding concepts for bipolar disorders and schizoaffective disorders. SNOMED is a standardized, multilingual vocabulary of clinical terminology that is used by physicians and other healthcare providers for the electronic exchange of clinical health information [22]. The ICD 9 and 10 codes included are listed in Additional file 1.

The outcome of interest was the development of treatment resistant depression (TRD) within 1 year after the first diagnosis of depression during pregnancy. There is no consensus definition of TRD, but one of the most common definitions requires a minimum of two prior treatment failures and confirmation of prior adequate dose and duration [13]. Implementing such a definition in claims databases is challenging because it is difficult to ascertain why medications were changed or stopped -- improvement, lack of efficacy or adverse events [23, 24]. Nonetheless, there is a definition of TRD to be used in claims database studies that has been validated [23]. Using this definition, TRD was defined as women with a diagnosis of depression who were dispensed 3 distinct antidepressants or 1 antidepressant and 1 antipsychotic in 1 year. Similar to the clinical definition of TRD, the ingredient of the antidepressant or antipsychotic is not germane. For example, a pregnant woman with depression who received sertraline, escitalopram and amitriptyline within a year after the diagnosis of depression is considered to have TRD. Similarly, a pregnant woman with depression who received sertraline and quetiapine within a year after the diagnosis of depression is considered to have TRD. This definition of TRD proved to be superior to definitions that attempted to define TRD based on adequacy of treatment dose and duration [23].

### Analysis

We calculated the proportion of pregnant women with incident or prevalent peripartum depression who went on to develop TRD. We described the population in terms of demographic characteristics and of medical conditions that have been associated with TRD [20]. The presence of these conditions was assessed in the year before the first diagnosis of depression during pregnancy and defined using SNOMED concepts.

We used chi square tests to assess whether differences of categorical variables were statistically significant and t-tests to assess whether differences of continuous variables were statistically significant. *P* values less than 0.05 were considered statistically significant.

The use of the CCAE database was reviewed by the New England Institutional Review Board (IRB) and was determined to be exempt, as this research project did not involve human subjects research.

To create the various cohorts, we used R, version 3.43. To make the statistical comparisons we used STATA SE, version 14.

## Results

**Fig. 2:**
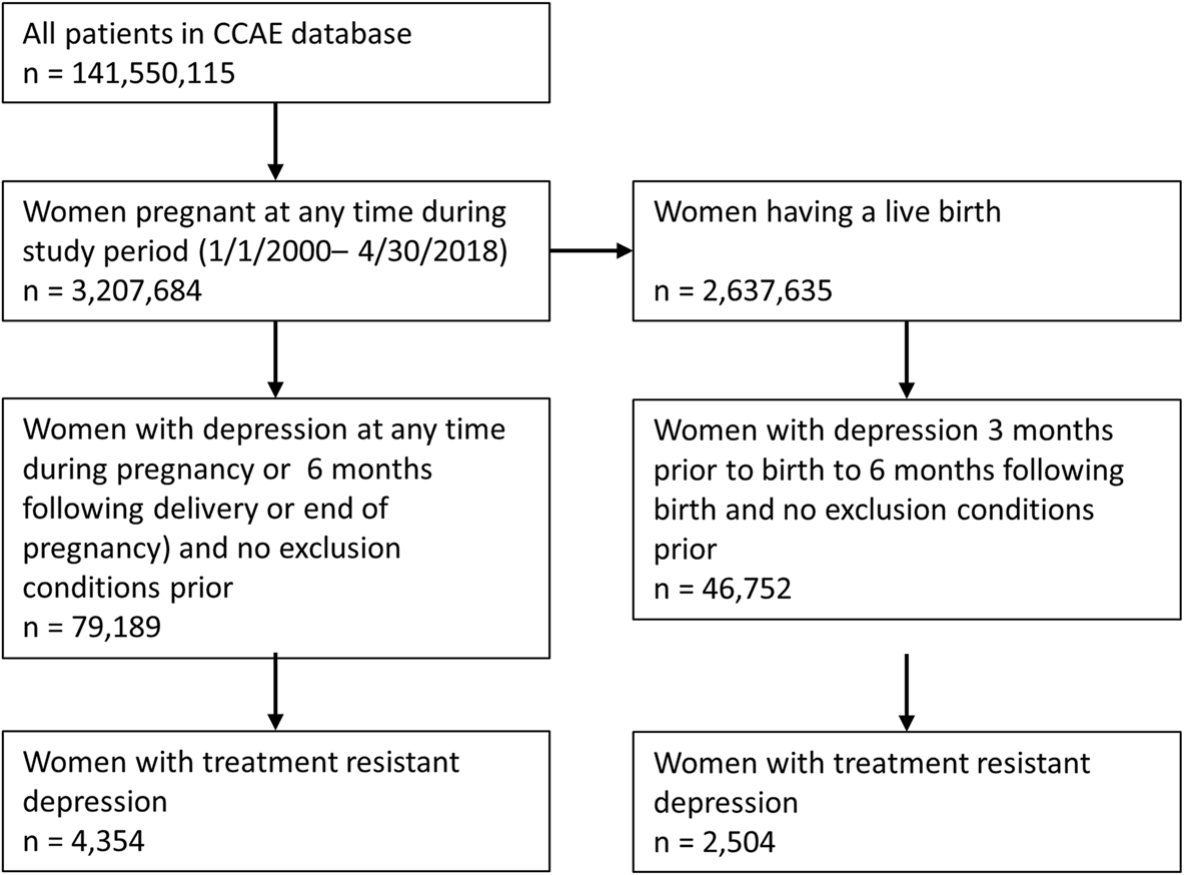
Flow of patient selection during study

The flow of patients in the study are depicted in Fig. 2.

Selective serotonin reuptake inhibitors (SSRIs), “other antidepressants” such as trazadone, and serotonin and norepinephrine reuptake inhibitors (SNRIs) were the most common classes of antidepressants used.

There were 3,207,684 pregnant women, of whom 2.47% were diagnosed with peripartum depression (Table 1). Of the women with peripartum depression, approximately half had incident depression during pregnancy.

**Table 1:** Incidence and prevalence of peripartum depression and treatment resistant depression (TRD) in pregnant women

	Number of pregnant women *N* = 3,207,684	Number of pregnant women giving live birth *N* = 2,637,365
Number with any peripartum depression	79,189	46,752
Number with TRD	4354	2504
Number with prevalent peripartum depression	38,733	25,164
Number with prevalent peripartum depression who developed TRD	2569	1535
Number with incident peripartum depression	40,456	21,588
Number with incident peripartum depression who developed TRD	1785	969

Of the women with peripartum depression, 5.50% developed TRD within 1 year of the peripartum diagnosis. The risk of developing TRD was 50% higher in women with prevalent depression (6.63%) than in women with incident peripartum depression (4.41%) (*P* < 0.0001).

Results were similar in women with peripartum depression diagnosed in the last trimester or during 6 months after a live delivery. Of these women, 5.36% developed TRD within 1 year of the peripartum diagnosis. The risk of developing TRD was also higher (36%) in women with prevalent depression (6.10%) than in women with incident peripartum depression (4.49%) (*P* < 0.0001).

Women who went on to develop TRD showed some differences from the women with peripartum depression who did not develop TRD. Women who went on to develop TRD had more comorbidities than women with prevalent or incident depression, including more, substance use disorders, anxiety, insomnia disorders, and more painful conditions, Table 2.

**Table 2:** Characteristics of pregnant women with incident or prevalent depression during pregnancy or up to 6 months after the end of pregnancy of pregnancy who did and did not develop treatment resistant depression

Characteristic	Pregnant women with depression but not TRD (*n* = 74,835)		Pregnant women with TRD (*n* = 4354)		*P* value
Age, years (mean, sd)	31.0	6.1	30.5	6.3	< 0.0001
Age group, years (n, %					
12–24	11,383	15.2%	780	17.9%	< 0.0001
25–34	42,113	56.3%	2387	54.8%	
35–44	20,647	27.6%	1141	26.2%	
45–56	692	0.9%	46	1.1%	
Deyo-Charlson severity index (mean, sd**)**	0.33	1.01	0.38	1.02	0.002
Comorbidities in prior year (n, %)					
Fatigue	527	0.7%	40	0.9%	0.115
Opioid dependence	427	0.6%	67	1.5%	< 0.0001
Drug abuse	371	0.5%	40	0.9%	< 0.0001
Tobacco dependence syndrome	2460	3.3%	200	4.6%	< 0.0001
Anxiety disorder	17,630	23.6%	1483	34.1%	< 0.0001
Obsessive-compulsive disorder	672	0.9%	87	2.0%	< 0.0001
Social phobia	127	0.2%	10	0.2%	0.345
Eating disorder	471	0.6%	42	1.0%	0.011
Insomnia	2192	2.9%	294	6.8%	< 0.0001
Pain of head and neck	14,504	19.4%	1107	25.4%	< 0.0001
Musculoskeletal pain	9354	12.5%	660	15.2%	< 0.0001

## Discussion

We found that approximately 2.5% of pregnant women had peripartum depression, and around 5.0% of women with peripartum depression developed TRD. The risk of developing TRD is higher (35 to 50%) in pregnant women with a history of depression. Women who developed TRD had more psychiatric comorbidities and more painful conditions than women who did not develop TRD.

The prevalence estimate of peripartum depression of 2.5% is close to the estimate provided by the American Psychiatric Association of 3% [4]. This estimate is much lower than published estimates of incidence or prevalence of peripartum depression, which range from 6.5 to 20% [6–8]. In those studies, to define depression, women responded to screening questionnaires or participated in a structured clinical interview according to DSM-IV or V criteria. In our study, peripartum depression was based on the presence of diagnosis codes, and these codes are part of a medical claim issued for reimbursement purposes not for research purpose. Therefore, it is expected that these codes are present in very symptomatic women or in women seeking care, so we are capturing only the more severe cases. Also, when the insurance does not have mental health coverage, these depression codes are not present in the claims databases. In addition, we included commercially insured women, a healthier population. Consequently, it is very likely that we are underestimating the risk of peripartum depression.

Five percent of women with peripartum depression developed TRD within 1 year. This is half the percentage found in the general population newly diagnosed with depression (10.4%) [20]. There is increasing evidence suggesting that women with peripartum depression are often untreated or suboptimally treated [25, 26] which could explain the lower rate of TRD observed in women with peripartum depression in this study (because the definition of TRD depends on treatment patterns). On the other hand, pregnant women are in frequent contact with healthcare providers during pregnancy because of recommended the antenatal care visits and therefore treatments/interventions can be implemented sooner and, if successful, could decrease the risk for women to develop TRD. In addition, there is evidence that non pharmacological approaches could also provide benefits similar to pharmacological treatments [27] and that these treatments may be more commonly used in women with peripartum depression. All these factors could contribute to the lower incidence of TRD observed in women with peripartum depression compared to the general population with depression.

History of depression has been identified as a risk factor for developing peripartum depression [6, 26, 28, 29] and discontinuing treatment for depression during pregnancy leads to a depression relapse during pregnancy more often than in women who maintain their medication [17]. We found that the risk of developing TRD was higher in women with a history of depression than in women with no history of depression, so this may also be a risk factor for developing TRD.

We found that women who went on to develop TRD suffered more frequently from substance use disorders, psychiatric conditions, insomnia, and pain than women with no TRD. These risk factors are in complete agreement with the risk factors for the general population with TRD [20]. Research suggests that there could be different types of perinatal depression, including one in which anxiety is a predominant symptom [30]. These different types of peripartum depression could respond differently to treatments [30]. Our study findings suggest that when anxiety is the predominant symptom in perinatal depression, similar to depression in the general population, it seems to be harder to treat [31].

A limitation of this study is that the TRD definition used is based, as many other definitions are, on lack of response to pharmacological treatments. This definition, ≥3 antidepressants or 1 antidepressant and ≥ 1 antipsychotics in a year, was found to be better at discriminating between subjects with and without TRD than a definition that included non-pharmacological treatments [23]. However, pregnant women were not the focus of the study, and in this segment of the population the use of nonpharmacological treatments may be more relevant than in the general population. The TRD definition used focused on the number of pharmacological treatments and dose was not germane. Although this definition was superior to the definitions that attempted to infer adequacy of treatment duration and dose, it is disparate to the clinical definition of TRD.

Another shortcoming is that we excluded individuals with bipolar disorders, these individuals when not responding to treatment often are prescribed not only antipsychotics but also mood stabilizers [32, 33] which are not included in the TRD definition. Therefore, our results can only be generalized to women with depression and not with bipolar depression.

As mentioned earlier, this study relies on the presence of codes to diagnose depression and the study population consisted of patients with commercial insurance. As a result, the population is younger and likely healthier compared to the general US population, and the results may not be generalizable to individuals enrolled in non-commercial health plans such as Medicaid and Medicare or uninsured. For example, it is known that lower socioeconomic status is a risk factor for peripartum depression [34] so in that segment of the population the prevalence of TRD could be higher.

There is an abundance of evidence regarding the negative impact of maternal depression on children, husbands/partners, and family, but it is not known how more deleterious it would be having perinatal depression that is treatment resistant [35, 36]. Further research is needed to understand the burden of peripartum depression that becomes TRD on women, their families and the society at large.

## Conclusions

This retrospective cohort study found approximately 5% of women with peripartum depression developed TRD, and the risk of developing TRD was higher in pregnant women with a history of depression. Women who developed TRD had more psychiatric comorbidities and more painful conditions than women who did not develop TRD.

## Additional file

Additional file 1: Codes used to define depression. (DOCX 13 kb)

## Data Availability

The data that support the findings of this study are available from IBM MarketScan® Commercial Database (CCAE) but restrictions apply to the availability of these data, which were used under license for the current study, and so are not publicly available.

## Abbreviations

CCAE: IBM MarketScan® Commercial Data
NDC: National Drug Codes
SNOMED: Systematized Nomenclature of Medicine-Clinical Terms
STAR*D: Sequenced Treatment Alternatives to Relieve Depression
TRD: Treatment resistant depression

## References

[1] Hahn-Holbrook J, Cornwell-Hinrichs T, Anaya I. Economic and Health Predictors of National Postpartum Depression Prevalence: A Systematic Review, Meta-analysis, and Meta-Regression of 291 Studies from 56 Countries. Front Psych. 2018;8(248). DOI: 10.3389/fpsyt.2017.00248. 10.3389/fpsyt.2017.00248 PMC5799244 29449816

[2] Eastwood J, Ogbo FA, Hendry A, Noble J (2017). Page A, for the Early Years Research G. The Impact of Antenatal Depression on Perinatal Outcomes in Australian Women. PLoS One.

[3] Halter MJ, Rolin-Kenny D, Grund F (2013). DSM-5: historical perspectives. J Psychosoc Nurs Ment Health Serv.

[4] American Psychiatric Association (2013). Unspecified Depressive Disorder. Diagnostic and Statistical Manual of Mental Disorders.

[5] Kendell RE, Chalmers JC, Platz C (1987). Epidemiology of puerperal psychoses. Br J Psychiatry.

[6] O’Hara MW, McCabe JE (2013). Postpartum depression: current status and future directions. Annu Rev Clin Psychol.

[7] Robertson-Blackmore E, Putnam FW, Rubinow DR, Matthieu M, Hunn JE, Putnam KT (2013). Antecedent trauma exposure and risk of depression in the perinatal period. J Clin Psychiatry.

[8] Gavin NI, Gaynes BN, Lohr KN, Meltzer-Brody S, Gartlehner G, Swinson T (2005). Perinatal depression: a systematic review of prevalence and incidence. Obstet Gynecol.

[9] Centers for Disease Control and Prevention (2017). Decline in Postpartum Depression. Jama.

[10] Ko JY, Rockhill KM, Tong VT, Morrow B, Farr SL (2017). Trends in postpartum depressive symptoms - 27 states, 2004, 2008, and 2012. MMWR Morb Mortal Wkly Rep.

[11] Russell JM, Hawkins K, Ozminkowski RJ, Orsini L, Crown WH, Kennedy S (2004). The cost consequences of treatment-resistant depression. J Clin Psychiatry.

[12] Berlim MT, Turecki G (2007). What is the meaning of treatment resistant/refractory major depression (TRD)? A systematic review of current randomized trials. Eur Neuropsychopharmacol.

[13] Gaynes BN, Asher G, Gartlehner G, Hoffman V, Green J, Boland E (2018). AHRQ Technology Assessments. Definition of Treatment-Resistant Depression in the Medicare Population.

[14] Rush AJ, Trivedi MH, Wisniewski SR, Nierenberg AA, Stewart JW, Warden D (2006). Acute and longer-term outcomes in depressed outpatients requiring one or several treatment steps: a STAR*D report. Am J Psychiatry.

[15] Conway CR, George MS, Sackeim HA (2017). Toward an evidence-based, operational definition of treatment-resistant depression: when enough is enough. JAMA Psychiat.

[16] Solberg LI, Engebretson KI, Sperl-Hillen JM, Hroscikoski MC, O'Connor PJ (2006). Are claims data accurate enough to identify patients for performance measures or quality improvement? The case of diabetes, heart disease, and depression. Am J Med Qual.

[17] Cohen LS, Altshuler LL, Harlow BL, Nonacs R, Newport DJ, Viguera AC (2006). Relapse of major depression during pregnancy in women who maintain or discontinue antidepressant treatment. Jama..

[18] Payne JL, Meltzer-Brody S (2009). Antidepressant use during pregnancy: current controversies and treatment strategies. Clin Obstet Gynecol.

[19] Daly EJ, Singh JB, Fedgchin M, Cooper K, Lim P, Shelton RC (2018). Efficacy and safety of intranasal Esketamine adjunctive to Oral antidepressant therapy in treatment-resistant depression: a randomized clinical TrialEfficacy and safety of intranasal Esketamine in DepressionEfficacy and safety of intranasal Esketamine in depression. JAMA Psychiat.

[20] Cepeda MS, Reps J, Ryan P (2018). Finding factors that predict treatment-resistant depression: results of a cohort study. Depress Anxiety.

[21] Matcho A, Ryan P, Fife D, Gifkins D, Knoll C, Friedman A (2018). Inferring pregnancy episodes and outcomes within a network of observational databases. PLoS One.

[22] Reich C, Ryan PB, Stang PE, Rocca M (2012). Evaluation of alternative standardized terminologies for medical conditions within a network of observational healthcare databases. J Biomed Inform.

[23] Cepeda MS, Reps J, Fife D, Blacketer C, Stang P, Ryan P (2018). Finding treatment-resistant depression in real-world data: how a data-driven approach compares with expert-based heuristics. Depress Anxiety.

[24] Fife D, Reps J, Cepeda MS, Stang P, Blacketer M, Singh J (2018). Treatment resistant depression incidence estimates from studies of health insurance databases depend strongly on the details of the operating definition. Heliyon..

[25] Cox EQ, Sowa NA, Meltzer-Brody SE, Gaynes BN (2016). The perinatal depression treatment Cascade: baby steps toward improving outcomes. J Clin Psychiatry.

[26] Flynn HA, Blow FC, Marcus SM (2006). Rates and predictors of depression treatment among pregnant women in hospital-affiliated obstetrics practices. Gen Hosp Psychiatry.

[27] Molyneaux E, Trevillion K, Howard LM (2015). Antidepressant treatment for postnatal depression. Jama..

[28] Drozdowicz-Jastrzebska E, Skalski M, Gdanska P, Mach A, Januszko P, Nowak RJ (2017). Insomnia, postpartum depression and estradiol in women after delivery. Metab Brain Dis.

[29] Suri R, Stowe ZN, Cohen LS, Newport DJ, Burt VK, Aquino-Elias AR (2017). Prospective longitudinal study of predictors of postpartum-onset depression in women with a history of major depressive disorder. J Clin Psychiatry.

[30] Putnam KT, Wilcox M, Robertson-Blackmore E, Sharkey K, Bergink V, Munk-Olsen T (2017). Clinical phenotypes of perinatal depression and time of symptom onset: analysis of data from an international consortium. Lancet Psychiatry.

[31] Ionescu DF, Niciu MJ, Richards EM, Zarate CA Jr. Pharmacologic treatment of dimensional anxious depression: a review. Prim Care Companion CNS Disord. 2014;16(3). 10.4088/PCC.13r01621 PMC4195641 25317369

[32] Hui Poon S, Sim K, Baldessarini RJ (2015). Pharmacological approaches for treatment-resistant bipolar disorder. Curr Neuropharmacol.

[33] Rybakowski JK (2010). Bipolar mood disorder and treatment-resistant depression. Ann General Psychiatry.

[34] Robertson E, Grace S, Wallington T, Stewart DE (2004). Antenatal risk factors for postpartum depression: a synthesis of recent literature. Gen Hosp Psychiatry.

[35] Burke L. (2003). The impact of maternal depression on familial relationships. International Review of Psychiatry.

[36] Cogill SR, Caplan HL, Alexandra H, Robson KM, Kumar R (1986). Impact of maternal postnatal depression on cognitive development of young children. Br Med J (Clin Res Ed).

