# Supplementary material for: Treatment resistant depression in women with peripartum depression
Source: BMC Pregnancy Childbirth. 2019 Sep 2;19:323. doi: 10.1186/s12884-019-2462-9 (PMC6721276; doi:10.1186/s12884-019-2462-9)
Supplement: Supplementary file 1 — Codes used to define depression. (DOCX 13 kb) [file 12884_2019_2462_MOESM1_ESM.docx]

**Supplementary file**

**Appendix 1**

Codes used to define depression

| F33.8 | Other recurrent depressive disorders |
| --- | --- |
| F32.3 | Severe depressive episode with psychotic symptoms |
| F32 | Depressive episode |
| F34.1 | Dysthymia |
| F92.0 | Depressive conduct disorder |
| F33.9 | Recurrent depressive disorder, unspecified |
| F33.1 | Recurrent depressive disorder, current episode moderate |
| F33 | Recurrent depressive disorder |
| F32.4 | Major depressive disorder, single episode, in partial remission |
| F33.42 | Major depressive disorder, recurrent, in full remission |
| F32.8 | Other depressive episodes |
| F20.4 | Post-schizophrenic depression |
| F33.3 | Recurrent depressive disorder, current episode severe with psychotic symptoms |
| F32.0 | Mild depressive episode |
| F48.0 | Neurasthenia |
| F32.2 | Severe depressive episode without psychotic symptoms |
| F33.41 | Major depressive disorder, recurrent, in partial remission |
| F32.1 | Moderate depressive episode |
| F33.40 | Major depressive disorder, recurrent, in remission, unspecified |
| F33.2 | Recurrent depressive disorder, current episode severe without psychotic symptoms |
| F33.0 | Recurrent depressive disorder, current episode mild |
| F33.4 | Recurrent depressive disorder, currently in remission |
| 300.4 | Dysthymic disorder |
| 296.33 | Major depressive affective disorder, recurrent episode, severe, without mention of psychotic behavior |
| 296.23 | Major depressive affective disorder, single episode, severe, without mention of psychotic behavior |
| 296.35 | Major depressive affective disorder, recurrent episode, in partial or unspecified remission |
| 311 | Depressive disorder, not elsewhere classified |
| 300.5 | Neurasthenia |
| 296.26 | Major depressive affective disorder, single episode, in full remission |
| 296.32 | Major depressive affective disorder, recurrent episode, moderate |
| 296.3 | Major depressive affective disorder, recurrent episode, unspecified |
| 296.31 | Major depressive affective disorder, recurrent episode, mild |
| 296.36 | Major depressive affective disorder, recurrent episode, in full remission |
| 296.34 | Major depressive affective disorder, recurrent episode, severe, specified as with psychotic behavior |
| 296.22 | Major depressive affective disorder, single episode, moderate |
| 296.3 | Major depressive disorder, recurrent episode |
| 296.21 | Major depressive affective disorder, single episode, mild |
| 296.25 | Major depressive affective disorder, single episode, in partial or unspecified remission |
| 296.2 | Major depressive affective disorder, single episode, unspecified |
| 296.2 | Major depressive disorder, single episode |
| 296.24 | Major depressive affective disorder, single episode, severe, specified as with psychotic behavior |
| F32.89 | Other specified depressive episodes |
| F32.81 | Premenstrual dysphoric disorder |
| F34.1 | Dysthymic disorder |
| F33.9 | Major depressive disorder, recurrent, unspecified |
| F33.8 | Other recurrent depressive disorders |
| F33.3 | Major depressive disorder, recurrent, severe with psychotic symptoms |
| F33.2 | Major depressive disorder, recurrent severe without psychotic features |
| F33.1 | Major depressive disorder, recurrent, moderate |
| F33.0 | Major depressive disorder, recurrent, mild |
| F32.8 | Other depressive episodes |
| F32.3 | Major depressive disorder, single episode, severe with psychotic features |
| F32.2 | Major depressive disorder, single episode, severe without psychotic features |
| F32.1 | Major depressive disorder, single episode, moderate |
| F32.0 | Major depressive disorder, single episode, mild |
| F33.4 | Major depressive disorder, recurrent, in remission |
| F33 | Major depressive disorder, recurrent |
| F32 | Major depressive disorder, single episode |
